# Correlation between incidental FDG PET/CT colorectal observations and endoscopic and histopathological results

**DOI:** 10.3892/ol.2013.1702

**Published:** 2013-11-25

**Authors:** TOVA RAINIS, ORIT KAIDAR-PERSON, DEAN KEREN, ALEXANDRA LAVY, ZOHAR KEIDAR

**Affiliations:** 1 Gastroenterology Unit, Bnai-Zion Medical Center, Haifa 31048, Israel; 2 Division of Oncology, Rambam Health Care Campus and Rappaport School of Medicine, Technion, Haifa 31096, Israel; 3 Department of Nuclear Medicine, Rambam Health Care Campus and Rappaport School of Medicine, Technion, Haifa 31096, Israel

**Keywords:** fluorodeoxyglucose positron emission tomography/computed tomography, incidental, colon, colonoscopy

## Abstract

Fluorodeoxyglucose positron emission tomography/computed tomography (FDG PET/CT) is used in the imaging workup of various malignancies. Incidental gastrointestinal observations on FDG PET/CT may be of clinical significance. The aim of the present study was to evaluate endoscopic and histopathological observations in patients referred for colonoscopy due to incidental FDG colonic uptake on a PET/CT study. Fifty-six patients with incidental colonic findings on FDG PET/CT underwent colonoscopy. Normal colonoscopies were observed in 63% of the patients. In 37% of the colonoscopies, we identified an endoscopic observation, including 67% with benign adenomatous polyps, 3% with hyperplastic polyps, 20% with advanced histological lesions and 10% with a malignancy.

## Introduction

Fluorodeoxyglucose positron emission tomography/computed tomography (FDG PET/CT) is used in the imaging workup of various malignancies for staging and surveillance (1). Incidental gastrointestinal observations are found in ~3% of patients undergoing evaluation for non-gastrointestinal diseases (2). These incidental observations in asymptomatic patients may be of clinical significance (2–4). The value of FDG PET or FDG PET/CT for identifying incidental pre-malignant and malignant colonic lesions has previously been reported (4–8). The early detection and treatment of colonic pre-malignant and malignant lesions may significantly improve survival, whereas further unnecessary studies may delay treatment of the primary disease. Studies that document the follow-up of these incidental observations to the point of histological confirmation are of great importance since they are the only means to evaluate the true positive rates of pre-malignant and malignant lesions identified by FDG PET/CT (8,9).

The aim of the present study was to evaluate endoscopic and histopathological observations in patients who were referred for colonoscopy due to incidental FDG colonic uptake on a PET/CT study.

## Patients and methods

The FDG-PET/CT database was retrospectively searched for patients in whom colonic FDG uptake was incidentally found and who then underwent colonoscopy between January 2007 and June 2009. Following identification of the specific patient population, the following data were retrieved from their medical records: Demographics, medical history, indications for performing FDG PET/CT, results of PET/CT and endoscopic and histopathological observations. Patients with known colon cancer or previous colonic surgery were excluded from further evaluation. The Institutional Review Board of Rambam Health Care Campus (Technion, Israel) approved this retrospective study and the requirement to obtain informed consent was waived.

## Results

**Figure 1:**
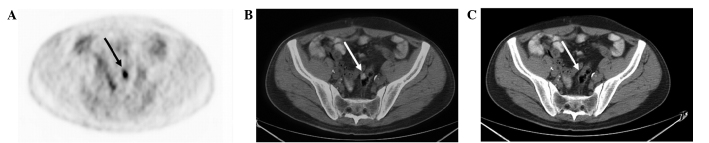
A 61-year-old male with a history of gastric carcinoma referred for suspicious pulmonary and liver findings on CT. (A) Transaxial FDG-PET demonstrates a focus of increased tracer uptake in the mid pelvis (arrow) localized by (B) PET/CT image to the rectosigmoid wall, which appears to be thickened on (C) CT. Following colonoscopy, a large polyp was resected, and the histological examination showed tubulovillous adenoma with moderate dysplasia. CT, computed tomography; FDG-PET, fluorodeoxyglucose-positron emission tomography.

**Figure 2:**
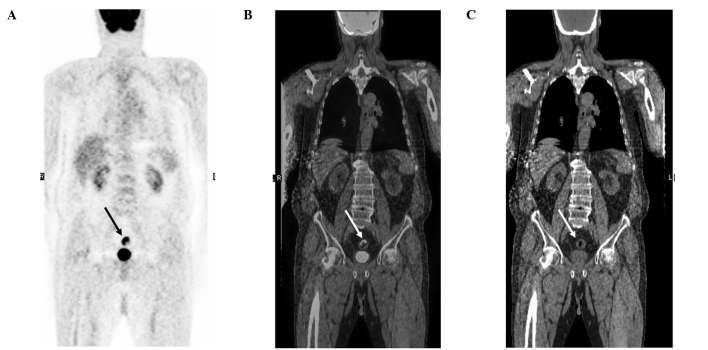
A 79-year-old male was referred for the assessment of a pulmonary nodule observed on CT. (A) Coronal FDG-PET, (B) PET/CT and (C) CT slices demonstrate intense FDG uptake in the proximal region of the colon (arrows). On colonoscopy, a large ulcer was found in 15 cm into the colon. The final diagnosis was of an adenocarcinoma of the colon. FDG PET/CT, fluorodeoxyglucose positron emission tomography/computed tomography.

The present study consisted of 56 patients (25 males and 31 females) with a mean age of 72 years old (range, 43–89 years old). The patient characteristics and indications for FDG PET/CT are listed in Table I. Overall, 60 foci of FDG uptake were found in the 56 patients. Of the occurrences indicative of focal FDG uptake in the colon, 13 were in the ascending colon, 6 in the transverse colon, 14 in the descending colon, 25 in the rectosigmoid and 2 in the ileum. The colonoscopy was normal in 35 patients (63%), and endoscopic observations were found in 21 patients (37%). In these 21 patients, 30 lesions were found; 20/30 (67%) were benign adenomatous polyps, 6/30 (20%) were advanced histological lesions (with features of high-grade dysplasia or carcinoma *in situ*), a malignancy was detected in 3/30 lesions (10%) and 1/30 (3%) was a benign hyperplastic polyp (Table II; Figs 1 and 2). Diverticular disease, with no endoscopic signs of inflammation, was found in 7/56 patients (12.5%) with positive FDG PET/CT results.

## Discussion

The potential of FDG PET or FDG PET/CT in colorectal screening has been studied in numerous trials (4–9). Focal colonic FDG uptake has up to a 70–80% probability of showing corresponding abnormal histopathological observations (4,6). Gutman *et al*(6) evaluated the positive predictive value of FDG PET/CT for the detection of colonic abnormalities. The study focused on the ability of FDG PET/CT to detect advanced adenomas (adenomas of >10 mm in diameter, adenomas with a villous component or moderate to severe dysplasia) and carcinomas. Among the 20 patients who underwent a colonoscopy, 5 had a normal colonoscopy (25%) and 15 (75%) patients had pathological lesions. In these 15 patients, a total of 18 lesions were found, including 2 benign hyperplastic polyps in the rectosigmoid and 16 advanced neoplasms. The advanced neoplasms consisted of 13 villous adenomas and 3 adenocarcinomas.

In an additional series of 3,210 PET scans performed for screening in asymptomatic patients, focal FDG avid uptakes were found in 20 patients, corresponding to 12 villous adenomas, 6 carcinomas and 2 tubular adenomas in the colonoscopy (4). These results are consistent with other studies showing that FDG PET ± CT is a sensitive tool to detect colonic premalignant lesions (4,6,7,9,10). However, caution must be applied, as the true negative rates are rarely evaluated in studies (9). It should be noted that FDG PET/CT is not a screening test for colon lesions; however, these were incidental observations detected during the evaluation of other diseases.

The majority of studies evaluating incidental colonic observations did not include patients with colorectal cancer. In the present study, the primary disease was colorectal cancer in 16 patients; however, all the patients had undergone surgical resection of their primary lesions and had no evidence of disease located in the bowel. Moreover, the medical files of patients who underwent a colonoscopy due to colonic observations on FDG PET/CT were reviewed, therefore, the rates of incidental colonic observations or the false negative rates were not evaluated. The true positive rates (36%) in the current study were lower than reported by others. Despite possible false-positive results, colonoscopy is recommended as the next diagnostic step for further evaluation of such observations. Since there are numerous confounding factors when evaluating the colorectal region, including fecal impaction, muscular peristaltic activity and inflammation, re-evaluation of the scan is advised. Gutman *et al*(6) indicated that in 3 patients, re-evaluation led to a different conclusion from that of the scan report regarding the nature of the FDG focal colonic uptake. The re-evaluation indicated that the colonic lesion was consistent with physiological activity, as the FDG uptake was located in the colonic lumen without bowel-wall involvement in an area of fecal stasis.

Although the present study found a lower incidence of lesions in the colon following colonoscopy, in 20 patients, colonic FDG observations matched the colonoscopic abnormalities, the majority of which required further surveillance and treatment. In the majority of cases of complete endoscopic resection, even in cases of carcinoma *in situ* or adenoma (tubular, tubulovillous or villous) with favorable histological features (T1 lesion, grade 1–2, no lymphovascular invasion, negative margins and no fragmentation of the specimen), no further treatment is required (11,12). However, appropriate endoscopic surveillance is mandatory (12).

In the present study, in one patient with a normal colonoscopy who exhibited a complete response to metastatic colon carcinoma, the colonic observation disappeared in a subsequent FDG PET/CT following chemotherapy. However, a later FDG PET/CT scan indicated diffuse metastatic disease involving the lung, liver and pelvis. A total of 35 FDG observations did not match any colonoscopic abnormality in the present study, thus, the PET/CT scan results were interpreted as false positive (62.5%). Of these false positive results, 7 revealed diverticular-disease with no endoscopic signs of inflammation. Peng *et al*(8) indicated that false-positive FDG uptake is more commonly observed in the right colon; this observation is not consistent with the present study. In the current study, the maximal standardized uptake (SUV_max_) was not evaluated and the clinical significance of the SUV_max_ from previous studies was inconclusive (5,8). The SUV_max_ value was higher in cancer patients, however, a high SUV_max_ value does not necessarily result in the detection of malignancies (5,8).

A positive FDG uptake that was followed by a normal endoscopy was considered physiological FDG uptake in the bowel. As shown in Table I, in specific patients, the indication for FDG PET/CT was pre-treatment staging, whereas for others, it was performed to evaluate the response to treatment of metastatic disease. Therefore, the timing and relevance of colonoscopic investigation must be dictated by clinical judgment and the status of the primary tumor.

## Figures and Tables

**Table I:** Indication for FDG PET/CT (n=56).

Primary disease	Patients, n	Indication for FDG PET/CT
Colorectal cancer	16	In 15 patients, imaging was performed as part of surveillance or due to increased levels of CEA. One patient had a known metastatic disease without bowel sites of disease
Non-Hodgkin lymphoma	14	Staging Assessment of new suspicious lesions
Breast cancer	5	Suspected metastatic disease
Lung cancer	7	Staging
Melanoma	3	Staging Response to systemic treatment
Hodgkin lymphoma	2	Surveillance
Bladder cancer	2	Assessment of new lung lesion Assessment of response to systemic treatment
Malignant hystiocytoma	1	Suspected recurrence
Gastric cancer	1	Suspected lesions in the lung and liver
Cervix cancer	1	Surveillance
Pancreatic cancer	1	Staging
Tongue cancer	1	Staging
Skin squamous cell cancer	1	Staging
Fever of unknown origin	1	To determine to source of prolonged fever

FDG PET/CT, fluorodeoxyglucose positron emission tomography/computed tomography; CEA, carcinoembryonic antigen.

**Table II:** Anatomical site of FDG foci and histological observations.

Parameter	Malignant, n	Advanced adenoma, n	Benign, n
Foci	3	6	21
Anatomical site			
Ascending colon		1	5
Descending colon	1	2	7
Rectosigmoid	2	3	9
Normal colonoscopy (including diverticular disease)			35

FDG, fluorodeoxyglucose.

## References

[1] Bar-Shalom R, Valdivia AY, Blaufox D (2000). PET imaging in oncology. Semin Nucl Med.

[2] Kamel EM, Thumshirn M, Truninger K (2004). Significance of incidental 18F-FDG accumulations in the gastrointestinal tract in PET/CT: correlation with endoscopic and histopathologic results. J Nucl Med.

[3] Agress H, Cooper BZ (2004). Detection of clinically unexpected malignant and premalignant tumors with whole-body FDG PET: histopathologic comparison. Radiology.

[4] Chen YK, Kao CH, Liao AC (2003). Colorectal cancer screening in asymptomatic adults: the role of FDG PET scan. Anticancer Res.

[5] Israel O, Yefremov N, Bar-Shalom R, Kagana O, Frenkel A, Keidar Z, Fischer D (2005). PET/CT detection of unexpected gastrointestinal foci of 18F-FDG uptake: incidence, localization patterns, and clinical significance. J Nucl Med.

[6] Gutman F, Alberini JL, Wartski M (2005). Incidental colonic focal lesions detected by FDG PET/CT. AJR Am J Roentgenol.

[7] Tatlidil R, Jadvar H, Bading JR, Conti PS (2002). Incidental colonic fluorodeoxyglucose uptake: correlation with colonoscopic and histopathologic findings. Radiology.

[8] Peng J, He Y, Xu J (2011). Detection of incidental colorectal tumours with 18F-labelled 2-fluoro-2-deoxyglucose positron emission tomography/computed tomography scans: results of a prospective study. Colorectal Dis.

[9] Weston BR, Iyer RB, Qiao W, Lee JH, Bresalier RS, Ross WA (2010). Ability of integrated positron emission and computed tomography to detect significant colonic pathology: the experience of a tertiary cancer center. Cancer.

[10] Drenth JP, Nagengast FM, Oyen WJ (2001). Evaluation of (pre-)malignant colonic abnormalities: endoscopic validation of FDG-PET findings. Eur J Nucl Med.

[11] Markowitz AJ, Winawer SJ (1997). Management of colorectal polyps. CA Cancer J Clin.

[12] Winawer SJ, Zauber AG, Fletcher RH (2006). Guidelines for colonoscopy surveillance after polypectomy: a consensus update by the US Multi-Society Task Force on Colorectal Cancer and the American Cancer Society. CA Cancer J Clin.

